# Estimation of the number and demographics of companion dogs in the UK

**DOI:** 10.1186/1746-6148-7-74

**Published:** 2011-11-23

**Authors:** Lucy Asher, Emma L Buckland, C Ianthi Phylactopoulos, Martin C Whiting, Siobhan M Abeyesinghe, Christopher M Wathes

**Affiliations:** 1 Royal Veterinary College, Hawkshead Lane, North Mymms, Hatfield, AL9 7TA, UK

## Abstract

**Background:**

Current estimates of the UK dog population vary, contain potential sources of bias and are based on expensive, large scale, public surveys. Here, we evaluate the potential of a variety of sources for estimation and monitoring of the companion dog population in the UK and associated demographic information. The sources considered were: a public survey; veterinary practices; pet insurance companies; micro-chip records; Kennel Club registrations; and the Pet Travel Scheme. The public survey and subpopulation estimates from veterinary practices, pet insurance companies and Kennel Club registrations, were combined to generate distinct estimates of the UK owned dog population using a Bayesian approach.

**Results:**

We estimated there are 9.4 (95% CI: 8.1-11.5) million companion dogs in the UK according to the public survey alone, which is similar to other recent estimates. The population was judged to be over-estimated by combining the public and veterinary surveys (16.4, 95% CI: 12.5-21.5 million) and under-estimated by combining the public survey and insured dog numbers (4.8, 95% CI: 3.6-6.9 million). An estimate based on combining the public survey and Kennel Club registered dogs was 7.1 (95% CI: 4.5-12.9) million. Based on Bayesian estimations, 77 (95% CI: 62-92)% of the UK dog population were registered at a veterinary practice; 42 (95% CI: 29-55)% of dogs were insured; and 29 (95% CI: 17-43)% of dogs were Kennel Club registered. Breed demographics suggested the Labrador was consistently the most popular breed registered in micro-chip records, with the Kennel Club and with J. Sainsbury's PLC pet insurance. A comparison of the demographics between these sources suggested that popular working breeds were under-represented and certain toy, utility and miniature breeds were over- represented in the Kennel Club registrations. Density maps were produced from micro-chip records based on the geographical distribution of dogs.

**Conclusions:**

A list containing the breed of each insured dog was provided by J. Sainsbury's PLC pet insurance without any accompanying information about the dog or owner.

## Background

The domestic dog (*Canis familiaris*) is among the most popular pets in Great Britain [1] and worldwide [2]. Currently, estimates of the dog population vary from 8 million (no confidence intervals provided [3]), to 10.5 million (95% CI: 9.6-11.4 million [4]), to 11.6 million (95% CI: 10.8-12.5 million [Adams VJ, Dean RS, Wills S: Pet ownership survey of UK households, unpublished data]). These estimates are based on large scale, costly public surveys undertaken at household level solely for the purposes of estimation. It may be that information already collected could be utilised to monitor the population of UK owned dogs as an alternative to large-scale public surveys.

Accurate estimates of the UK dog population and canine demographics are of interest to a wide range of stakeholders including: animal welfare charities, pet product manufacturers, pet care providers, insurance companies, veterinarians, researchers and the Government. An understanding of the size, dynamics, demographics and geographical distribution of the population of dogs in the UK is important for research into canine disease [5] and welfare surveillance [6,7]. Dogs can act as vectors for zoonotic disease [8,9] and might be important in the transmission of pathogens from wildlife to humans. Demographic factors such as breed, gender, age [10,11], and neuter status [12] can influence the incidence of canine disease, and age and neuter demographics can facilitate prediction of population dynamics [13,14].

To date, and to the authors' best knowledge, attempts to estimate the population of dogs in the UK, or areas of the UK, have used only public surveys conducted by telephone [4,15] or the postal service [Adams VJ, Dean RS, Wills S: Pet ownership survey of UK households, unpublished data]. The most recent peer-reviewed findings [4] used factors found to affect dog ownership, scaled up to population-level using the 2001 census, to estimate the owned UK dog population. An annual public survey is conducted by the Pet Food Manufacturing Association (PMFA) to obtain yearly estimates of the size of the UK dog population (PMFA, personal communication), although the exact method used for sampling and estimation is not available publically. Public surveys are necessarily large to cover the population of over 25 million households in the UK [16], and are therefore costly e.g. Murray et al. [4] involved 13,795 telephone calls. Response biases can exist, e.g. non-pet owners or non-pet lovers would be expected to be less likely to respond.

Records maintained by veterinary practices and practice managements could be a useful source for estimating the number of (companion) dogs in the UK. The small number of veterinary practices (approximately 3700; Royal College of Veterinary Surgeons, RCVS), relative to the number of households in the UK, means that surveys of veterinary practices are less costly and could cover a higher percentage of the population of interest. To date, veterinary practices have been under-utilised in acquiring data about dog populations, and their use has been limited to demographics rather than estimation. One study in Switzerland considered the demographics of the dog population using data collected from veterinary practices amongst other sources [17]. Lund et al. [5] collected data from 52 veterinary practices in the USA to build a profile of the age, breed, sex, body condition score, and diet of dogs and cats and recently used data from 651 practices to perform a retrospective analysis of castrations in dogs and cats [18].

In the UK, there are approximately 20 pet insurance companies. Data from insurance companies in Sweden have been used successfully to gain extensive knowledge of the Swedish population of dogs and could be a useful source of information on the UK canine population, for example, studies of the risk factors for dystocia [19], heart disease [20] and diabetes [21], and survival rates and mortality of various breeds have been calculated [22-25]. A comparison between the population of insured dogs and the general dog population in Sweden, suggested that insured dogs were representative of the general population [26]. An estimated 78% of dogs are insured in Sweden [27] compared with between 20 and 24% in the UK [[27], Sainsbury's pet insurance: personal communication, 2009]. Thus data from UK insurance companies would encompass a smaller proportion of the UK population than equivalent data from Swedish insurance companies.

Micro-chipping databases are a further potential source of information as they contain information including: owner or keeper's full name; two telephone numbers; address and postcode; breed; sex; year or date of birth; colour; description or identifying marks; and significant medical problems or medication required [28]. There are three micro-chipping databases in the UK; Petlog (chip brand names: Allflex, Bayer, Datamars, Fit and Fertile, Jecta, Pet I, Peddymark Ltd, PetCode, Pet-detect and CoreRFID), Pettrac (chip brand name: Avid) and Anibase (chip brand names: Identichip, Virbac and PetCode). Of a sample of American dogs which had been lost and returned to their owners, 8% had a micro-chip [29]. A separate study of dogs re-homed from American rescue centres found 42.3% were micro-chipped [30].

The UK Kennel Club publishes freely available pedigree dog registrations by breed each year. These statistics have previously been used to make inferences about the pedigree population of dogs, including trends in the numbers of particular breeds [31-33].

The Pet Travel Scheme (also known as PETS) was introduced by the Government in February 2000 to allow movement of certain animals between certain countries without prolonged quarantine. DEFRA publish annual statistics about the number of dogs that enter the UK under the Pet Travel Scheme, which have the potential to be used as a source of information on the UK dog population.

Here we evaluate the utility of different sources in the estimation of companion dog population and in producing reliable information on the demographics of this population. We consider the following sources: 1) a public survey; 2) a survey of veterinary practices; 3) pet insurance company registrations; 4) micro-chip registrations; 5) Kennel Club registrations; 6) the Pet Travel Scheme registrations.

To produce estimates of the population of dogs from various subpopulations, we modified a method loosely based on capture-mark-recapture approaches used in field studies. True capture- mark-recapture methods, which have previously been applied to free-roaming or stray dogs [34,35], require identification of individuals captured and recaptured, which was not feasible here. In the current study, a conceptual framework was used; subpopulations of dogs (based on sources 2,3 and 5) were 'captured' using one source, then 'recaptured' using a public survey, by questioning owners about whether or not their dog(s) belonged to each one of the subpopulations of interest. Using this method, we were also able to elucidate the relationships between the subpopulations of dogs covered by each of these sources. We used Bayesian statistics to combined estimations of populations and subpopulations. Bayesian statistics can be used to indicate the likelihood of an item falling within a certain category or range of values.

## Methods

### Veterinary practices

The RCVS list was used to contact all practices with valid email addresses. These practices were invited by email to complete a questionnaire in May 2009 and non-responders were sent a reminder in July 2009. The questionnaire asked about the number of dogs registered at the practice(s) and postcode(s) for the relevant practices. The email was followed up by an identical postal survey of selected practices in July 2009. 350 practices were contacted by letter using every tenth practice on the (alphabetically-ordered) RCVS list.

The RCVS list was matched against National Statistics Postcode Directory [34] and information concerning geographic location, country and urban-rural classification was obtained. A postcode location was considered to be: (1) Urban, if the census output area (COA) or general register for Scotland (GROS) fell within an area with a population of 10,000 people or more; (2) Town or Fringe, if the COA/GROS fell within this category for England and Wales, the Small Town or Intermediate Settlement category for Northern Ireland, or the Small Town categories for Scotland; (3) Village, if the COA/GROS fell within this category for England and Wales, the Village for Northern Ireland, or the Rural Accessible Settlement categories for Scotland; (4) Hamlet and Isolated Dwelling, if the COA/GROS fell within this category for England and Wales, the Small Village, Hamlet or Open Countryside category for Northern Ireland, or the Remote Rural categories for Scotland.

### Pet insurance companies

Attempts were made to contact all insurance companies covering UK dogs and the Association of British Insurers by email, telephone and letter. Details of insured dogs by breed and by county were provided by J. Sainsbury's PLC pet insurance. A single estimate of the subpopulation of insured dogs of 2 million (without confidence intervals) was provided by the Association of British Insurers.

### Micro-chip registrations

Attempts were made to contact all micro-chip companies registering UK dogs. Anonymous micro-chip registrations were obtained from the Anibase database on Identichip, Petcode and Virbac micro-chips. The dates for the micro-chip registrations were not provided and therefore microchip registrations were used to examine only canine demographics and geographical distribution. The micro-chip data provided the number of registrations by breed and county in separate databases. Data were matched where possible such that each breed entry represented a breed recognised by the Kennel Club [35], a crossbreed or mongrel, or was labelled unknown; and each county entry represented a county or combination of counties and/or unitary authorities recognised by the Office of National Statistics, or was described as unknown and excluded from further analysis. Due to lack of specificity in many breed names, it was necessary to collapse certain breeds into one breed group, for instance, the Toy, Miniature and Standard poodle, were labelled Poodle. Due to lack of specificity in county names certain counties and unitary authorities were combined, for instance East and West Sussex were labelled Sussex. Due to the commercial nature of the micro-chip data, we present percentages of dogs of each breed registered and percentage of dogs from each county or unitary authority area, rather than exact figures. Data from the Office of National Statistics concerning the area (km 2) and population were used to adjust percentages of dogs in each county for the area and population and were used to produce county-based maps of the density of the population of companion dogs.

### Kennel Club registrations

Kennel Club breed registration statistics from 1999 to 2008 were obtained. The Kennel Club records new registrations but does not require dogs to be de-registered when they die. Therefore, to calculate the size of the sub-population of dogs registered with the Kennel Club, we applied survival curves [25] (which plot the number animals that remain living as a function of time) to the registration data. Confidence intervals (95%) were estimated using the variance in the probability of survival of all breeds covered by Egenvall et al. [25].

### The Pet Travel Scheme

Information on the number of pet dogs entering the UK under the Pet Travel Scheme was accessed from the Defra website [36]. The Pet Travel Scheme information did not contain records of animals leaving the UK under the Pet Travel Scheme and concerned a relatively small proportion of animals. Thus we decided not to attempt to use these data further.

### Public survey

The public survey was conducted by telephone in July and August 2009 by Royal Veterinary College staff following approval by the Royal Veterinary College Ethics & Welfare committee. Phone numbers were obtained from 17 British Telecom telephone directories, selected at random. A quota sampling system was employed such that 30 valid calls from each phonebook were used for analysis. A pseudo-random protocol was developed to reduce bias in the telephone numbers selected. The survey consisted of five simple questions concerning pet dog ownership in the household. Participants were asked question 1 below followed by questions 2-5 unless their answer to question 1 was zero in which case no further questions were asked.

1) how many dogs they owned

2) the number of dogs they owned that had travelled abroad

3) the number of dogs they owned that were registered with a veterinary practice

4) the number of dogs they owned that were insured

5) the number of dogs they owned that were UK Kennel Club registered.

Other potential sources, pet food manufacturers, major retailers and veterinary pharmaceutical industries, were considered but were discounted from the study because of difficulties during initial investigations in obtaining accurate, reliable data that are often held confidentially.

### Statistical Analysis

Statistical analysis was conducted in R 2.11.0 [37].

To examine possible biases in the responses from veterinary practices, we analysed response or non-response against country and urban-rural classification using χ^2 ^tests. To establish the distribution in the number of dogs registered at veterinary practices, we used visual inspection, Maximum Likelihood estimation and the Kolmogorov-Smirnov test and the R package mixdist [38].

To calculate estimations of the proportion of dogs in each subpopulation, the size of certain subpopulations, and the size of the UK owned-dog population, we used a Bayesian approach. First estimates of the proportion of dogs in the subpopulations were made using posterior predictive distributions:

PREDICTED SUBPOPULATION (1 = in subpopulation, 2 = not in subpopulation)

~ Benoulli probability (Pi)

Logit(Pi)= α(1)

where α is the regression intercept and Pi is the probability of a dog from the entire population of dogs to be in the subpopulation of dogs in question. The model was repeated for dogs in three subpopulations: registered with a veterinary practice, insured and Kennel Club registered. It was estimated using data sampled from the responses of the public survey. An estimate of dog numbers in the veterinary subpopulation was based on an intercept-only model with the intercept based on the distribution shown in equation 2 sampled from the responses of veterinary practices on their numbers of registered dogs, multiplied by 2550 (the number of veterinary practices in the UK, 3638 divided by 1.32, the mean number of practices represented by a single response to the survey). The subpopulation for the insured dogs was fixed at 2 million according to the estimate provided by the Association of British Insurers and the subpopulation of Kennel Club registered dogs was drawn from a normal distribution with mean and variance, derived from the application of survival curves to the number of kennel club registrations. Estimates of the total population of owned dogs were calculated based on each estimate of the subpopulation divided by the proportion of dogs in that subpopulation. In the case of the public survey estimate a normally distributed intercept-only model sampled from the number of dogs per household in each area multiplied by the number of households in Britain (25.9 million [16]). Non informative priors were used with the exception of the estimates of the dog population which used Murray et al.'s [4] estimate and variance. Models were fitted using a Markov Chain Monte Carlo simulation based on Gibbs sampling in the R package rjags 2.1.0-5 [39]. Such Bayesian approaches produce posterior distributions of the modelled parameters which we present here as median and 95% Credibility Intervals. The Bayesian model was analysed in the R package coda [40]. After assessment for convergence, we based results on 100,000 iterations using 2 Markov Chain Monte Carlo chains, a burn in of 10,000 iterations, and a thinning interval of 50.

## Results

### Veterinary practices

**Figure 1:**
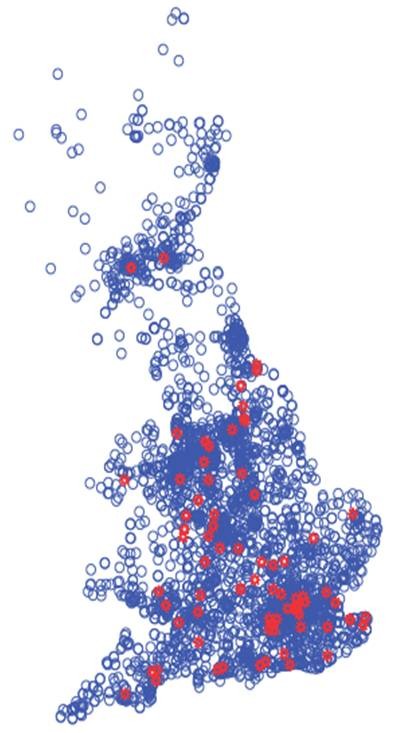
**Geographical distribution of RCVS-listed veterinary practices in England, Scotland and Wales**. Practices which responded to the survey of registered dog numbers are shown in red.

A total of 2763 out of a possible 3638 veterinary practices were contacted. We received valid responses from 103 veterinary practices across the UK giving an overall response rate of 3.7%. A certain proportion of responses covered more than one practice listed in the RCVS database: the mean ± SE number of practices represented per response was 1.32 ± 0.2. Obtaining responses from veterinary practices was affected by country of residence (χ2 = 1.84, df = 5, p = 0.037) with a disproportionate number of responses from England, few responses from Scotland and Wales and no responses from Northern Ireland, the Channel Islands or the Isle of Man (Table 1). The urban-rural classification did not affect the responses (χ2 = 1.07, df = 3, p = 0.785). The geographical distribution of all veterinary practices in England, Wales and Scotland from the RCVS records, indicating those that responded to the survey is presented in Figure 1.

**Table 1:** Profile of RCVS-listed veterinary practices and practices which responded to the survey.

		Total listed in RCVS database	Number of responses (% of RCVS database)
**Country**	England	2985	72 (2.41%)
	Northern Ireland	90	0
	Scotland	334	2 (0.59%)
	Wales	203	3 (1.47%)
	Isle of Man	8	0
	Channel Islands	13	0

**Urban-Rural Classification**	Urban	2546	58 (2.27%)
	Town or Fringe	675	13 (1.92%)
	Village	231	3 (1.29%)
	Hamlet and Isolated Dwelling	160	3 (1.87%)

**Figure 2:**
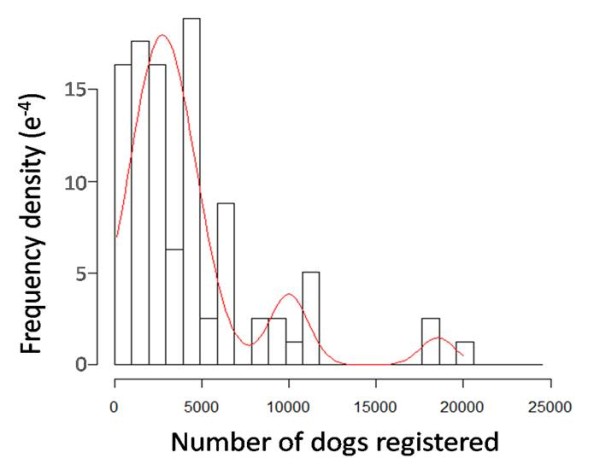
**The observed (black) and fitted (red) distribution of the number of dogs registered at veterinary practices**. Observed values are based on the survey of veterinary practices (N = 77).

The number of dogs registered at veterinary practices ranged from 100 to 28,356. A mixture distribution comprising of three normal (Gaussian) distributions was found to best fit the observed distribution of the number of dogs registered at veterinary practices (see Figure 2) as follows:(2)

where *z Ɲ (a,b) *is a Normal distribution with mean *a*, standard deviation *b *and a weighting of *z*.

### Pet insurance companies, Kennel Club and micro-chip registrations

The Association of British Insurers provided an estimate of 2 million insured dogs in the UK. The number of UK dogs currently registered with the Kennel Club was estimated to be 2,116,871 (95% CI: 1,696,647-2,425,060).

Some micro-chip registration data was available for a total of 157 of the 210 breeds recognised by the Kennel Club and 132 breeds were covered by Sainsbury's pet insurance (see Table 2 for a summary of the top 20 ranked breeds). For dogs insured with J Sainsbury's PLC pet insurance, 2.73% were classified as pedigree breed unknown and 28.91% of dogs were crossbreeds. For 23.37% of micro-chipped dogs, the breed was unknown and a further 12.60% of dogs were registered as crossbreeds.

**Table 2:** Popularity of breeds ranked in the top 20 registrations from micro-chip records, J Sainsbury's PLC pet insurance and UK Kennel Club registrations.

Breed	% micro-chip registered dogs	% dogs insured with J Sainsbury's PLC	% of KC registrations	Micro-chip Breed popularity rank*	J Sainsbury's PLC pet insurance popularity rank*	KC popularity rank (2008)	% increase in KC registrations (1999-2008)
Unknown	23.37	2.73					
Cross	12.60	28.91					
Retriever (Labrador)	9.96	10.89	16.40	1	1	1	35.44
Staffordshire Bull Terrier	4.92	3.75	3.90	2	4	6	8.53
German Shepherd Dog (Alsatian)	4.22	3.60	4.32	3	5	4	-33.52
Border Collie	4.21	2.88	0.86	4	8	26	25.59
Spaniel (Cocker)	3.49	5.65	8.16	5	2	2	68.25
Spaniel (English Springer)	3.39	2.92	5.40	6	7	3	20.07
Yorkshire Terrier	2.49	1.70	1.43	7	10	17	-46.16
Retriever (Golden)	2.48	3.54	3.32	8	6	7	-28.05
West Highland White Terrier	2.48	2.34	2.66	9	9	10	-49.16
Cavalier King Charles Spaniel	2.01	4.83	4.07	10	3	5	-3.03
Rottweiler	1.57	0.79	0.95	11	20	23	-50.41
Border Terrier	1.46	1.61	3.32	12	11	8	138.84
Shih Tzu	1.25	1.25	1.99	13	12	11	45.33
Boxer	1.04	0.89	2.67	14	16	9	-25.68
Lhasa Apso	0.86	0.68	1.86	15	23	14	61.22
Poodle	0.83	0.88	1.31	16	17	21	-0.66
Bichon Frise	0.70	1.08	1.00	17	13	22	13.27
Greyhound	0.63	0.33	0.02	18	39	163	17.95
Weimaraner	0.63	0.91	0.83	19	15	27	-4.75
Dalmatian	0.60	0.76	0.57	20	22	37	-40.99
Miniature Schnauzer	0.60	1.00	1.93	21	14	13	142.19
Dachshund	0.60	0.57	1.98	22	26	12	1.47
Bulldog	0.54	0.78	1.65	23	21	15	115.72
Chihuahua	0.53	0.39	2.57	25	32	18	-18.80
Bull Terrier	0.49	0.79	1.06	27	19	20	11.02
Whippet	0.48	0.26	1.21	28	44	19	104.80
Pug	0.29	0.59	1.62	37	25	16	524.83
King Charles Spaniel	0.28	0.83	0.07	38	18	105	-15.38

* excluding unknown and crossbreeds

The most popular breeds according to micro-chip registrations were: the Labrador retriever, Staffordshire Bull Terrier, German Shepherd Dog, Border Collie and Cocker Spaniel. The most popular breeds according to J Sainsbury's PLC pet insurance were: Labrador retriever, Cocker Spaniel and Cavalier King Charles Spaniel. The most popular breeds according to Kennel Club registrations were: Labrador retriever, Cocker Spaniel, English Springer Spaniel, Staffordshire Bull Terrier and German Shepherd Dog.

Some breeds were over-represented in the micro-chip registrations as compared with the 2008 Kennel Club registrations including Greyhounds, Border Collies, Dalmatians, Rottweilers and Yorkshire Terriers. Breeds over-represented in the 2007 Kennel Club registrations as compared to the Micro-chip registrations included Pugs and Dachshunds. Disparity between J Sainsbury's PLC pet insurance breed ranks and Kennel club registrations were notable in the Greyhound and the King Charles Cavalier Spaniel.

**Figure 3:**
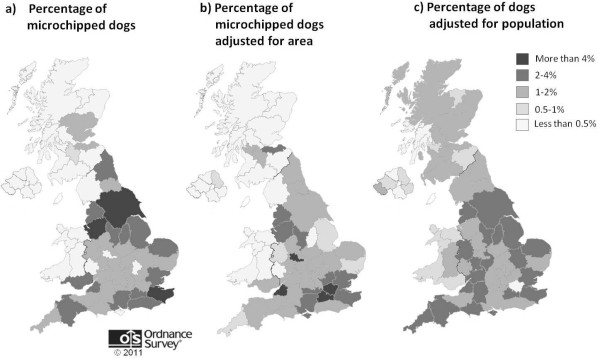
**Geographical distribution of micro-chipped dogs by county**. Map produced with permission from Ordnance Survey.

The counties with the highest percentage of micro-chipped dogs on the database accessed (the Anibase database on Identichip, Petcode and Virbac micro-chips) were: Yorkshire (10.33%), Kent (5.34%) and Cheshire (5.2%) (Figure 3). Twenty-one counties contained fewer than 0.5% of micro-chipped dogs. When the percentage of micro-chipped dogs was adjusted for the size of the area, Avon (14.89%), the West Midlands (9.89%) and Surrey (4.07%) had the highest percentage of micro-chipped dogs and seventeen counties had less than 0.5% of micro-chipped dogs. The percentage of micro-chipped dogs was more even between counties when controlled for the human population: no counties had more than 4% of the micro-chipped dogs and four counties had less than 0.5%.

### Public survey

In the public survey a total of 1656 phone calls were made; we achieved a response rate of 37% giving a total of 614 valid responses. A quota sample of 30 valid responses for each telephone directory area was used, such that results are based on 510 responses. We found a total of 181 dogs in 122 dog-owning households with 23.92% of households owning at least one dog and 1.47 ± 0.02 dogs per dog-owning household (Table 3). Of the dogs surveyed (and prior to application of Bayesian estimation), 71.82% were vet-visiting (95% CI: 55-100%), 2.76% were registered with the Pet Travel Scheme (95% CI: 0-33%), 40.33% were insured (95% CI: 29-100%), and 30.39% were registered with the Kennel Club (95% CI: 11-69%).

**Table 3:** Summary of results from the public survey by telephone directory area, prior to Bayesian estimation.

Phonebook area	Response rate	Number of dog owning households out of 30	Number of dogs	Percentage of households with a dog (%)	Mean dogs (± SE) per dog owning household
Aberdeen and Shetland	35.33	12	14	23.00	1.17 ± 0.05
Bath and West Wiltshire	29.70	8	10	27.00	1.25 ± 0.06
Blackpool and the Fylde	31.91	4	7	13.00	1.80 ± 0.26
Bromley and Orpington	38.46	3	3	10.00	1.00 ± 0.00
Central and South Wales	35.58	20	29	67.00	1.45 ± 0.06
Cornwall and Isles of Scilly	65.22	10	13	33.00	1.33 ± 0.19
Durham and Wearside	37.04	4	7	13.00	1.30 ± 0.05
Glasgow South	31.25	7	14	23.00	1.75 ± 0.24
Hemel Hempstead	60.00	5	7	17.00	2.00 ± 0.32
Huddersfield	51.72	5	7	17.00	1.40 ± 0.11
Liverpool	40.67	7	7	23.00	1.40 ± 0.18
Manchester Central	44.12	3	5	10.00	1.00 ± 0.00
Milton Keyes	37.04	6	11	20.00	1.67 ± 0.19
Nottingham	31.91	8	9	27.00	1.83 ± 0.19
Salisbury	33.71	8	16	27.00	1.00 ± 0.00
Stratford-upon-Avon	34.88	6	15	20.00	1.00 ± 0.00
Walsall, Cannock and Lichfield	30.41	6	7	20.00	2.50 ± 0.53
**Total**	**37.08**	**122**	**181**	**23.92**	**1.47 ± 0.02**

For each area a quota of 30 calls were made.

### Combining sources

**Figure 4:**
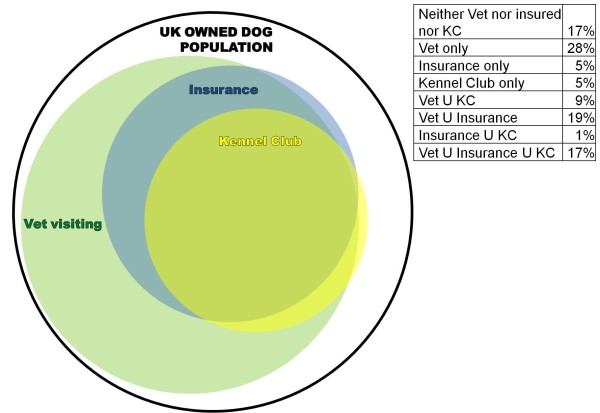
**Venn diagram and summary table of relationships between sources based on public survey (before Bayesian estimation)**. Vet stands for registered with a veterinary practice, KC stands for Kennel Club registered and U stands for union, e.g., 'Vet U KC' indicates dogs that are both registered with a veterinary practice and the Kennel Club.

The percentage (before Bayesian estimation) of dogs in the public survey that were veterinary registered, had pet insurance and were Kennel Club registered and all the permutations of these factors were used to create a Venn diagram of the relations between the sources and therefore subpopulations of dogs (Figure 4).

**Figure 5:**
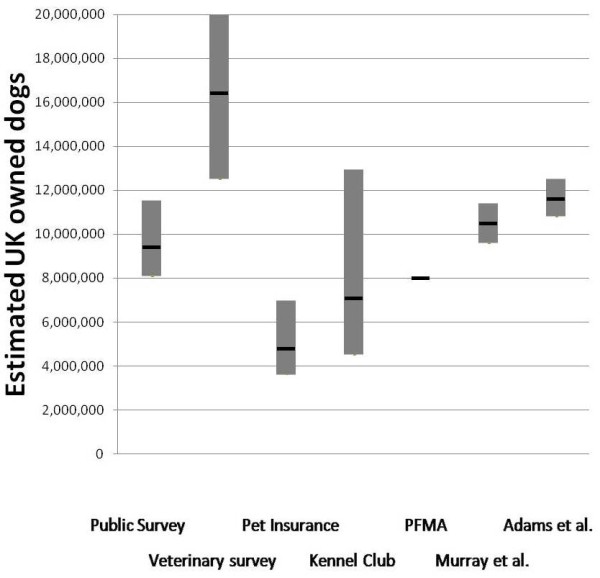
**Comparison of estimates from sources used in the current study, (median and 95% Credibility Intervals for the Public survey, and Public survey and Veterinary survey, Pet Insurance and Kennel Club) and other recent estimates with Confidence Intervals where available (PFMA, 2009, Murray et al., 2010 and Adams et al., unpublished)**.

A Bayesian model was used to calculate estimates and Credibility Intervals for the proportion of dogs in each subpopulation of interest and of the total population of owned dogs based on each of the subpopulation figures and proportions (see Table 4). Total population estimates are compared against existing estimates in Figure 5.

**Table 4:** Estimates (Median and Credibility Intervals) based on Bayesian analysis of the Public survey and the estimates of the subpopulations of dogs from each source.

	Public survey	Veterinary Practices	Pet Insurance	UK Kennel Club
Estimate of population	9,409,000	16,400,000	4,774,000	7,069,000

95% CI	8,089,000-11,530,000	12,510,000-21,470,000	3,609,000-6,973,000	4,514,000-12,930,000

Proportion of dogs in sub population		0.77	0.42	0.29

95% CI		0.62-0.92	0.29-0.55	0.17-0.43

Estimate of subpopulation		8,283,869	2,000,000*	2,116,871*

95% CI		7,813,432-11,225,435	NA	1,696,647*-2,425,060*

*indicates figures not based on Bayesian analysis where CI refers to Confidence Intervals

## Discussion

### Population estimates

We produced four estimates of the UK owned-dog population using combinations of the public survey with other information sources. We believe our population estimate based on the survey of veterinary practices to be an overestimate. It is expected that many veterinary practices do not de-register dogs when they are no longer part of the practice's clientele and therefore that some dogs may be registered with more than one practice. Practice surveys could still have utility in estimating the dog population, provided that the proportion of overestimation remains constant over time and could be controlled for in calculations. The response rate from veterinary practices was low, which may be because the requested information on the number of dogs registered at a practice was not readily available. The increase in computerised systems may facilitate this in the future.

The lowest estimate of the dog population was produced using the estimate of the insured dog subpopulation provided by Association of British Insurers. It is reasonable to assume that the subpopulation estimate of insured dogs was underestimated. It could be that the insured subpopulation estimate provided of 2 million was an underestimate or alternatively that percentage of dogs that were reported as insured from the public survey may have been inaccurate. Previously the UK insurance penetration has been estimated at 24% of dogs (personal communication [26,27]). The estimate based on the subpopulation of Kennel Club registered dogs was more similar to other estimates; however, the precision of this estimate was low. One source of bias that may have affected our public survey is that of owner responsibility. If more responsible dog owners were also more likely to participate in our survey then these results will be biased towards responsible owners who may be more likely to own insured and vet- visiting dogs

### Demographics

As might have been anticipated, popular working breeds, such as Greyhounds and Border collies were ranked lower in the Kennel Club registrations compared with the insurance and micro-chip sources. Some of the differences in breed popularity could have reflected the differences in the time period and age of dogs covered by these sources. This is supported by the observation that breeds that have decreased in the number of Kennel Club registrations over the past 10 years were also found to be less popular in Kennel Club registrations compared with other sources. Popular toy, utility or miniature breeds were more prevalent according to Kennel Club registrations as compared with the other sources, e.g. Dachshund, Pug, Bulldog and Chihuahua. The figures presented here on the percentage of purebred dogs are likely to be biased with a disproportionate number of pedigree or purebred dogs insured or micro-chipped as compared to crossbreeds.

Examining the geographical distribution of micro-chipped dogs from the Anibase database, we found that most dogs were located in Yorkshire, Cheshire and Kent. When controlled for area, Avon, the West Midlands and Surrey had the highest percentages of dogs. These counties also have large human populations, and indeed when we controlled for the human population, the percentages of micro-chipped dogs were more evenly spread across the counties. One notable exception was the Greater London area, where fewer dogs were registered on the micro-chip database than might be expected from the size of the human population. The geographical distribution of veterinary practices from the RCVS list appeared to follow the pattern of human population density. It is not known whether there are any geographical biases in the propensity for owners to micro-chip their dogs.

### Evaluation of sources

Public surveys are necessary for a true understanding of the entire dog population, but they must be purposely conducted, are expensive, time consuming and can be biased. Estimates from other sources used here represent biased samples; however, by recognising, understanding and accounting for such bias, some of the sources could generate accurate estimates of the dog population at comparatively low cost.

Our survey of veterinary practices had a very low response rate, less than 4%, with veterinary practices in England over-represented. The distribution of the number of dogs registered was best fitted with a mixture distribution, suggesting that we were detecting different groupings in terms of size, perhaps with some responses being from practice managers and others from individual practices. This study indicates that the majority of companion dogs are veterinary-registered. Surveys of veterinary practices could be useful in estimating the dog population in the future although this would be best achieved if there were first research undertaken to understand the distribution and methods of data storage used by practices in the UK.

Only one of approximately twenty insurance companies provided us with information about insured dogs, possibly due to the financial sensitivity of this information. We were not able to obtain data on the market share of insurance companies and therefore we could not use the data provided by J Sainsbury's PLC to make estimates about the UK population of companion dogs. Dogs insured with this company are not expected to be representative of all insured dogs. As data from Swedish pet insurance companies [19-26] have demonstrated, insurance databases can be useful for studies of the pet population, particularly epidemiological studies. Information provided by pet insurers could, perhaps, be best employed to understand trends in disease, for example, J Sainsbury's PLC pet insurance company has published figures about breed related claims [41].

The primary purpose of canine micro-chip databases is reunification of lost dogs with their owners [28]. Perhaps for this reason, there was little standardisation in the manner in which breeds and counties were reported in the micro-chip database we used. We were unclear as to the dates of registration that the database covered and whether dogs that were deceased had been removed from the database. Microchip databases can, however, be useful sources for demographic and geographical distribution on the UK dog population.

Kennel Club breed registrations are freely available, produced annually, have been recorded over many decades and (according to our estimates) represent approximately one third of the dog population. With a greater understanding of how this subpopulation varies with the entire population, it could be a useful source for interim approximate estimates of the UK dog population.

The Pet Travel Scheme was not a useful source for estimating the population of UK dogs, since there were no corresponding records of animals leaving the UK. Furthermore, less than 3% of dogs in the public survey had travelled abroad using the Pet Travel Scheme.

## Conclusions

With the exception of the Pet Travel Scheme, all sources investigated in this study had some utility in understanding aspects of the UK population of companion dogs.

## Authors' contributions

LA conceived of and assisted with study design and data collection, performed statistical analyses and wrote the first draft of the manuscript. ELB assisted in co-ordinating data collection, provided edits and comments on funding body report and manuscript. CIP assisted in study design and data collection and commented on the manuscript. MCW devised the veterinary questionnaire. SMA assisted in data collection, provided edits and comments on funding body report and manuscript. CMW assisted in data collection, provided edits and comments on funding body report and manuscript. All authors read and approved the final manuscript.

## References

[B1] Westgarth C Pinchbeck GL Bradshaw JW Dawson S Gaskell RM Christley RM Factors associated with dog ownership and contact with dogs in a UK community BMC Vet Res 2007 3 5 10.1186/1746-6148-3-5 17407583 PMC1852100

[B2] Toribio JA Norris JM White JD Dhand NK Hamilton SA Malik R Demographics and husbandry of pet cats living in Sydney, Australia: results of cross-sectional survey of pet ownership J Feline Med Surg 2009 11 6 449 461 10.1016/j.jfms.2008.06.010 19070524 PMC7130031

[B3] PFMA How many dogs and cats are there in the UK? http://www.pfma.org.uk/statistics Accessed: 21/10/2010

[B4] Murray JK Browne WJ Roberts MA Whitmarsh A Gruffydd-Jones TJ Number and ownership profiles of cats and dogs in the UK Vet Rec 2010 166 6 163 168 10.1136/vr.b4712 20139379

[B5] Lund E Armstrong P Kirk C Kolar L Klausnor J Health status and population characteristics of dogs and cats examined at private veterinary practices in the United States J Am Vet Med Assoc 1999 214 1336 1341 10319174

[B6] Bateson P Independent Inquiry into Dog Breeding 2010

[B7] Collins L Asher L Summers J Diesel G McGreevy P Welfare epidemiology as a tool to assess the welfare impact of inherited defects on the pedigree dog population Anim Welf 2010 19 S1 67 75

[B8] Westgarth C Pinchbeck GL Bradshaw JW Dawson S Gaskell RM Christley RM Dog- human and dog-dog interactions of 260 dog-owning households in a community in Cheshire Vet Rec 2008 162 14 436 442 10.1136/vr.162.14.436 18390853

[B9] Robinson RA Pugh RN Dogs, zoonoses and immunosuppression J R Soc Promot Health 2002 122 2 95 98 10.1177/146642400212200210 12134775

[B10] Egenvall A Bonnett BN Olson P Hedhammar A Gender, age, breed and distribution of morbidity and mortality in insured dogs in Sweden during 1995 and 1996 Vet Rec 2000 146 18 519 525 10.1136/vr.146.18.519 11321213

[B11] Egenvall A Bonnett BN Shoukri M Olson P Hedhammar A Dohoo I Age pattern of mortality in eight breeds of insured dogs in Sweden Prev Vet Med 2000 46 1 1 14 10.1016/S0167-5877(00)00135-5 10854932

[B12] Bryan JN Keeler MR Henry CJ Bryan ME Hahn AW Caldwell CW A population study of neutering status as a risk factor for canine prostate cancer Prostate 2007 67 11 1174 1181 10.1002/pros.20590 17516571

[B13] Baldock FC Alexander L More SJ Estimated and predicted changes in the cat population of Australian households from 1979 to 2005 Aust Vet J 2003 81 5 289 292 10.1111/j.1751-0813.2003.tb12577.x 15084040

[B14] Di Nardo A Candeloro L Budke CM Slater MR Modeling the effect of sterilization rate on owned dog population size in central Italy Prev Vet Med 2007 82 3-4 308 313 10.1016/j.prevetmed.2007.06.007 17692414

[B15] Downes M Canty MJ More SJ Demography of the pet dog and cat population on the island of Ireland and human factors influencing pet ownership Prev Vet Med 2009 92 1-2 140 149 10.1016/j.prevetmed.2009.07.005 19700212

[B16] Office for National Statistics Labour Force Survey 2009 London: Office for National Statistics

[B17] Horisberger U Stark KD Rufenacht J Pillonel C Steiger A Demographic characteristics of dog population in Switzerland Schweiz Arch Tierheilkd 2004 146 5 223 232 10.1024/0036-7281.146.5.223 15185459

[B18] Trevejo R Yang M Lund EM Epidemiology of surgical castration of dogs and cats in the United States J Am Vet Med Assoc 2011 238 7 898 904 10.2460/javma.238.7.898 21453178

[B19] Bergstrom A Nodtvedt A Lagerstedt AS Egenvall A Incidence and breed predilection for dystocia and risk factors for cesarean section in a Swedish population of insured dogs Vet Surg 2006 35 8 786 791 10.1111/j.1532-950X.2006.00223.x 17187641

[B20] Egenvall A Bonnett BN Haggstrom J Heart disease as a cause of death in insured Swedish dogs younger than 10 years of age J Vet Intern Med 2006 20 4 894 903 10.1111/j.1939-1676.2006.tb01803.x 16955814

[B21] Fall T Hamlin HH Hedhammar A Kampe O Egenvall A Diabetes mellitus in a population of 180,000 insured dogs: incidence, survival, and breed distribution J Vet Intern Med 2007 21 6 1209 1216 10.1111/j.1939-1676.2007.tb01940.x 18196728

[B22] Bonnett BN Egenvall A Age patterns of disease and death in insured Swedish dogs, cats and horses J Comp Pathol 2010 142 S1 S33 38 19932895 10.1016/j.jcpa.2009.10.008

[B23] Bonnett BN Egenvall A Hedhammar A Olson P Mortality in over 350,000 insured Swedish dogs from 1995-2000: I. Breed-, gender-, age- and cause-specific rates Acta Vet Scand 2005 46 3 105 120 10.1186/1751-0147-46-105 16261924 PMC1624819

[B24] Bonnett BN Egenvall A Olson P Hedhammar A Mortality in insured Swedish dogs: rates and causes of death in various breeds Vet Rec 1997 141 2 40 44 10.1136/vr.141.2.40 9253830

[B25] Egenvall A Bonnett BN Hedhammar A Olson P Mortality in over 350,000 insured Swedish dogs from 1995-2000: II. Breed-specific age and survival patterns and relative risk for causes of death Acta Vet Scand 2005 46 3 121 136 10.1186/1751-0147-46-121 16261925 PMC1624818

[B26] Sallander M Hedhammar A Rundgren M Lindberg JE Demographic data of a population of insured Swedish dogs measured in a questionnaire study Acta Vet Scand 2001 42 1 71 80 10.1186/1751-0147-42-71 11455903 PMC2202336

[B27] Agira International Pet Insurance Animal insurance and the veterinary profession- The Swedish example Federation of Veterinarians of Europe: General Assembly Meeting 2009 Stockholm

[B28] BSAVA Micro-chip Advisory Group Micro-chip advisory group (MAG) code of practice for databases holding permanent identification data for dogs http://www.bsava.com/Advice/MicrochipAdvice/tabid/154/Default.aspx Accessed: 15/10/2010

[B29] Lord LK Wittum TE Ferketich AK Funk JA Rajala-Schultz PJ Search and identification methods that owners use to find a lost dog J Am Vet Med Assoc 2007 230 2 211 216 10.2460/javma.230.2.211 17223753

[B30] Lord LK Reider L Herron ME Graszak K Health and behavior problems in dogs and cats one week and one month after adoption from animal shelters J Am Vet Med Assoc 2008 233 11 1715 1722 10.2460/javma.233.11.1715 19046028

[B31] Asher L Diesel G Summers J McGreevy P Collins L Inherited defects in pedigree dogs. Part 1: Disorders related to breed standards Vet J 2009 182 3 402 411 10.1016/j.tvjl.2009.08.033 19836981

[B32] Mannhart T A catch-neuter-release project for free-roaming dogs and cats in Rhodes, Greece: Problem analysis and effectiveness of the strategy 2007 University of Bern

[B33] World Health Organization Second WHO consultation on oral immunization of dogs against rabies Geneva 1990 6

[B34] UKBORDERS UK Postcode Directories. Quarterly Directory for August 2009. Edina Census 2009

[B35] The Kennel Club Breed registration statistics http://www.thekennelclub.org.uk/item/1128 Accessed: 25/10/2009

[B36] DEFRA PETS: Statistics: Number of pet cats and dogs entering the UK under PETS each month http://archive.defra.gov.uk/wildlife-pets/pets/travel/pets/procedures/stats.htm Accessed: 23/07/2010

[B37] The R Core Development Team R: A Language and Environment for Statistical Computing Version 2.11.1 http://www.R-project.org Accessed: 31/05/2010

[B38] MacDonald P Finite Mixture Distribution Models R package version 05-2 2008

[B39] Plummer M rjags: Bayesian graphical models using MCMC R package 210-5 2010

[B40] Plummer M Best N Cowles K Vines K Coda: output analysis and diagnostics for MCMC R package version 013-4 2009

[B41] Sainsbury's press release UK Pet Lovers Make Nearly 200,000 Insurance Claims For Their Cats and Dogs http://www.24-7pressrelease.com/press-release/uk-pet-lovers- make-nearly-200000-insurance-claims-for-their-cats-and-dogs-123597.php Accessed: 10/11/2009

